# Effects of proxy solicitation and director ownership on directors’ career consequences

**DOI:** 10.1016/j.heliyon.2023.e14471

**Published:** 2023-03-11

**Authors:** Tai-Wei Zhang, Yun-Chi Lee

**Affiliations:** a Department of Finance, Ming Chuan University, Taipei, Taiwan; b Department of Business Administration, Soochow University, Taipei, Taiwan

**Keywords:** Proxy solicitation, Director elections, Director reputation, Busy directors, Shareholder voice

## Abstract

This study investigates how proxy solicitation and director ownership jointly affect directors' career consequences in Taiwan. We report that assent votes partly arising from proxies without shareholder voice increase the likelihood of departure for directors with higher ownership in firms soliciting proxies, especially for busy directors. Since proxy votes do not build extra reputation, this generates no spillover effect for both non-busy and busy directors. Overall, we support the arguments based on prior studies that votes holding information on shareholder voice have implications on directors’ careers. Furthermore, different board seats provide unequal incentives for busy directors.

## 1 Introduction

In this paper, we explore the joint effects of proxy solicitation and director ownership on the career consequences of directors. The motivation for this investigation stems from a special context of proxy solicitation in Taiwan. In practical operation, shareholders have the fundamental right to elect directors at a shareholder meeting to represent their interests. The Company Act in Taiwan prescribes a three-year term for directors, thereby leading to some boards having to be elected in specific years, mostly in June. In light of the ownership structure of listed companies being relatively dispersed, proxy solicitation is a prevalent practice to reach the quorum required to hold an annual meeting and obtain board seats successfully. If a quorum is not reached, the meeting may be held at a second call. The incumbent directors or controlling shareholders often use corporate funds to solicit proxies for facilitating shareholder meetings and director positions sponsored by management. To attain their board seats, director candidates take advantage of their own shareholdings or controlling shareholders and proxy votes if focal firms with election events solicit proxies. Therefore, it does not seem likely that directors will enhance their career prestige if they are elected through proxy solicitation, because proxy voting fails to convey the voice of stockholders.

Compared to prior related literature using dissent votes to examine directors' career consequences, our study contributes to the literature by using the viewpoint of assent votes arising from proxy solicitation to address it. In the labor market for directors, reputation is a crucial disciplining device [[1], [2], [3], [4], [5], [6]]. Dissent votes in uncontested director elections signal negative sentiment among shareholders, directly affecting directors' turnover and the opportunity to obtain additional outside director board seats [7]. This means that shareholder voice implied in votes impacts directors' career consequences. In contrast, proxy solicitation refers to a mute voice from a shareholder's viewpoint, implying that assent arising from proxy votes is incapable of preserving and enhancing the reputation in the labor market for directorships.

Generally speaking, directors with good reputations become busy and serve on multiple boards. However, not all directorships are the same for a director. Different board seats hold different values, and some directorships are more prestigious than others. Once faced with a trade-off, directors usually relinquish lower ranked directorships, presumably to avoid the negative reputational effects, compared with higher ranked ones [6]. This phenomenon may be obvious for busy directors. In addition to proxy solicitation, director shareholding is the key to obtaining a directorship. Therefore, we expect that assent votes partially resulting from proxies increase the likelihood of departures for directors with higher shareholdings at firms soliciting proxies in the subsample of busy directors, but not in the subsample of non-busy directors. Because proxy votes do not build extra reputation, assent votes received by proxies are expected to generate no spillover effect for both non-busy and busy directors.

To verify our conjunction, we employed a sample of 1671 director elections held by Taiwan-listed companies between 2015 and 2020, consisting of 9568 observations of elected directors. “Busy directors” are defined as directors who hold at least three directorship seats at different publicly traded firms, while “non-busy directors” are those with at least one but fewer than three directorship seats at different publicly traded firms [8,9]. The results show that the interaction term between proxy solicitation and director ownership has positive effects on assent votes for both non-busy and busy directors. In addition, we find that directors with higher ownership at firms soliciting proxies tend to leave the directorship at the focal firm only for busy directors. That is, directors with large shareholdings at a firm soliciting proxies consider that the position contributes less to their reputation and is, therefore, less preferable, particularly for busy directors compared to non-busy directors. The evidence is consistent with arguments by Masulis and Mobbs (2014) that busy directors rank their directorships and allocate their limited resources by distributing more effort and resources to firms where their directorships are considered more prestigious or valuable [6]. As proxy votes involve no shareholder voice, we find no effect of the interaction term between proxy solicitation and director ownership on the net changes in external directorships for both non-busy and busy directors. Regarding the endogeneity concern, our results remain robust to two-stage least squares (2SLS) and propensity score matching (PSM).

This study is structured as follows: Section 2 addresses the literature review and develops hypotheses; Section 3 describes the sample and outlines the research design; Section 4 discusses the empirical results, and Section 5 presents conclusions.

## 2 Related literature and hypotheses development

In this section, we review related literature on directors’ reputations and career consequences, further developing our hypotheses.

Prior studies show that in addition to financial incentives [10,11], reputation is a significant incentive for board directors as it directly involves the building of personal prestige and the likelihood of seeking new board positions [1,12,13]. For example, outside directors may be motivated by reputation concerns to exit companies in order to avoid being associated with any negative publicity generated [[14], [15], [16]]. Gow et al. (2018) argue that directors manage their reputation through disclosure choices in which information is withheld about directors’ associations with troubled firms [17]. On the other hand, a vast amount of literature examines how the good performance and decision-making of directors help to build their reputation. Studies show that favorable career outcomes follow “good” performance, such as the termination of underperforming CEOs [18], rescission of takeover defenses [19], receipt of high takeover premiums [20], and general firm performance [21]. Mirror-image examples demonstrate how poor performance—such as accounting restatements [22], shareholder lawsuits [8], option backdating [23], and proxy contest nominations [24]—leads to fewer opportunities in the director labor market.

Generally, directors with good reputations are in high demand and serve on additional board seats as rewards. Directors who serve on multiple boards become busy. Busy directors, however, are most criticized for not effectively monitoring management [25], suggesting that busy directors are weak in enhancing firm value [[26], [27], [28], [29]]. To avoid the above phenomenon, directors who serve on multiple directorships may actively manage their reputations by valuing each directorship differently based on the relative reputational benefits a board offers [6]. This is because not all directorships are the same for a director. Different board seats hold different values, and some directorships are more prestigious than others. Masulis and Mobbs (2014) indicate that considering limited time and energy, directors with multiple appointments distribute their efforts depending on a board's relative contribution to a director's reputation [6]. Therefore, busy directors are more concerned about their reputation and are likely to choose appointments that will enhance their reputation.

In spite of early literature indicating that shareholders have limited ability to vote out directors in uncontested elections [30,31], a number of recent empirical studies show that shareholder voice as expressed through votes in director elections is related to director careers, meaning that shareholder activism in the form of voting stimulates directors to establish a reputation as a diligent monitor. For example, Fos et al. (2018) point out that more effective monitoring will be implemented by directors since the election approaches [32]. Zhang (2021) reports that the risk of being removed by shareholders motivates directors to adopt corporate policies that align the interests of shareholders [33]. Aggarwal et al. (2019) show that shareholder dissent votes in uncontested director elections have power and reflect the negative sentiment of a firm's owners about the relevant director, negatively affecting director career consequences [7]. Specifically, shareholder dissent votes are associated with increased director turnover and fewer outside director board seats. Directors receiving expressions of dissent feel greater pressure regarding career concerns, such as managerial positions or directorships in other firms, potentially becoming unsuitable candidates for other positions. Taken as a whole, votes in director elections serve as an informational channel regarding the director market and contribute toward the functioning of the reputation channel, indicating that shareholder voice implied in votes impacts directors' career consequences.

Unlike previous literature using shareholder voice of dissatisfaction with directors [7], our study employs proxy solicitation referring to a mute voice from a shareholder's viewpoint to examine the importance of shareholder voice on directors' career consequences. It is intuitive that director ownership is positively associated with the proportion of assent votes acquired by the director, irrespective of proxy solicitation by the focal firm, for both non-busy and busy directors. If higher assent votes arise partially from proxy votes involving no shareholder voice, there is no additional reputation built for directors, thereby expecting that such directors increase the likelihood of departures, especially when they are busy. Differing from shareholder dissent votes representing a strong negative signal for directors, proxy votes just involve no shareholder voice so that no spillover effect on other outside director board seats is generated for both non-busy and busy directors. The above arguments lead to our two hypotheses:

Hypothesis 1 The interaction term between proxy solicitation and director ownership has a positive effect on director turnover for busy directors, but not for non-busy directors.

Hypothesis 2 The interaction term between proxy solicitation and director ownership has no effect on increasing the future number of directorships held at other firms for both non-busy and busy directors.

## 3 Research methodology

### 3.1 Data

We obtained a sample of firms listed on the Taiwan Stock Exchange (TWSE) that held a director election at the annual general meeting from 2015 to 2020. Finance-related institutions (Code 28) were excluded. Our sample includes only those elected directors who faced an election in a particular year. We followed Aggarwal et al. (2019) to exclude directors identified as executives at the time of the elections to focus on supervisory or outside directors [7]. We manually identified information indicating the solicitation of proxy voting rights in an election from a free database maintained by the Securities and Futures Bureau of the Financial Supervisory Commission (http://free.sfi.org.tw). We hand-collected data on the voting outcomes of each elected director from the Market Observation Post System of the TWSE Corporation, which were available since 2015. That is why we chose 2015 as the start of our sample period. The Taiwan Economics Journal (TEJ) database is the primary source for board data (number of directorships) and individual director-specific features (director's sex). The TEJ provides data on the service history and biographical data of individuals who serve as directors of Taiwan-listed companies and on firms' characteristics.

### 3.2 Research model

The primary empirical model is specified as follows:

$$Y_{i,j}=\beta_{0}+\beta_{1}\times {Proxy}_{i}\times {DirOwn}_{i,j}+\beta_{2}\times {Proxy}_{i}+\beta_{3}\times {DirOwn}_{i,j}$$

(1) $$+\beta_{4}\times F_{i}+\beta_{5}\times D_{i,j}+\varepsilon_{i,j}$$

The unit of observation is director *j* facing an election at firm *i*. *Y* is one of the following measures: assent votes (*%Assent* and *Excess%Assent*), director turnover (*Departure*), and changes in outside directorships (*Chg_Dirship_t* and *Chg_Dirship_t+1*). All variables are defined in the Appendix. *Proxy* × *DirOwn* is an interaction term of interest, where *Proxy* is a binary variable indicating whether a firm solicits proxies in the director election and *DirOwn* is the shareholdings of each elected director at the time of the election. We refer to previous research to include controls that are considered relevant for a director’ career consequences [7,24]. The control variables include firm characteristics denoted by vector *F* and director-specific characteristics denoted by vector *D*. Firm-level controls include firm size (*Size*), stock returns (*StockRet*), return on assets (*ROA*), and institutional ownership (*InstOwn*). Director-level controls include director tenure (*Tenure*), gender (*Male*), and education (*MasterAbv*). We use logistic regressions to estimate equation (1) when the dependent variable is *Departure*, otherwise ordinary least squares (OLS) regressions are employed. All specifications include industry fixed effects. *T*-statistics are based on standard errors corrected for heteroscedasticity and clustered at the firm level.

## 4 Empirical results

### 4.1 Descriptive statistics

Table 1 reports the annual frequency of our sample, which consists of 1671 director election events and involves 9568 elected directors. Because directors are appointed for a three-year term, about one-third of listed companies hold director elections every year. Due to more and more companies being listed on the TWSE year by year, our sample firms and their respective elected directors are increasing in a three-year round, i.e., comparing the sample size in year 2015 versus 2018, year 2016 versus 2019, and year 2017 versus 2020. However, there is a downward trend in the percentage of firms soliciting proxies, from 42.64% in 2015 to 36.15% in 2020. Nonetheless, over one-third of the firms holding director elections solicit proxies. On average, approximately 40.04% of firms make proxy solicitation in director elections, while 38.72% of elected directors belong to firms soliciting proxies.

**Table 1:** Year distribution of the sample.

Year	# of firms	Firm-level % of proxy solicitations	# of elected directors	Director-level % of proxy solicitations
2015	258	0.4264	1,272	0.4096
2016	277	0.4440	1,341	0.4407
2017	280	0.4536	1,377	0.4539
2018	272	0.3934	1,527	0.3739
2019	288	0.3299	2,041	0.3248
2020	296	0.3615	2,010	0.3652
Total	1,671	0.4004	9,568	0.3872

*Notes:* This table reports the annual firm- and director-level sample size of the director elections held between January 2015 and December 2020.

Table 2 reports the descriptive statistics of the variables. Table 2(A) shows that the average percentage of votes received by an elected director is 87.65%. On average, each director acquires 2.45% fewer votes than the mean assent votes at the focal firm holding an election. An individual director holds a mean of around three outside directorships. The average increase in the number of outside directorships over the election year and the next year is about 0.21 to 0.30. Table 2(B) shows that the mean director ownership is 4.44%. Table 2(C) provides firms' and directors’ characteristics.

**Table 2:** Summary statistics for the sample of directors.

	N	Mean	SD	Q3	Median	Q1
**Panel A: Voting outcomes and career consequences**						
%Assent	9,568	0.8765	0.3031	0.9968	0.9046	0.7523
Firm%Assent	9,568	0.9011	0.1140	0.9791	0.9375	0.8496
Excess%Assent	9,568	−0.0245	0.2851	0.0569	−0.0106	−0.0834
Departure	9,566	0.0175	0.1310	0.0000	0.0000	0.0000
Dirship_t-1	9,217	3.1107	5.6078	4.0000	1.0000	0.0000
Dirship_t	9,568	3.2938	5.4775	4.0000	2.0000	0.0000
Dirship_t+1	4,277	3.1337	4.9674	4.0000	2.0000	0.0000
Chg_Dirship_t	9,217	0.2141	2.0973	0.0000	0.0000	0.0000
Chg_Dirship_t+1	4,224	0.2981	2.7905	1.0000	0.0000	0.0000
**Panel B: Independent variables**						
Proxy	9,568	0.3872	0.4871	1.0000	0.0000	0.0000
DirOwn	9,568	0.0444	0.1051	0.0287	0.0007	0.0000
**Panel C: Control variables**						
*Firm characteristics*						
Size	9,568	16.2259	1.6116	16.9496	15.9394	15.1425
StockRet	9,568	0.0847	0.3515	0.2130	0.0322	−0.1125
ROA	9,568	0.0884	0.0835	0.1315	0.0779	0.0399
InstOwn	9,568	0.4836	0.2309	0.6644	0.4931	0.2987
*Director characteristics*						
Tenure	9,568	11.1496	11.0791	16.8300	7.4200	2.8300
Male	9,568	0.8676	0.3390	1.0000	1.0000	1.0000
MasterAbv	9,568	0.5629	0.4961	1.0000	1.0000	0.0000

*Notes:* This table reports summary statistics for elected directors at the director elections in shareholder meetings held between January 2015 and December 2020. Panels A, B, and C present the measurements of voting outcomes and career consequences, independent variables, and control variables at the director level. All variables are defined in the Appendix.

### 4.2 Regression analysis

Table 3 presents the results of votes received by elected directors and career consequences for non-busy and busy directors. The results regarding voting outcomes are presented in Models (1) to (2) and (6) to (7). Models (1) and (6) use *%Assent* as the dependent variable, while Models (2) and (7) use *Excess%Assent* as the dependent variable. We find that the coefficients of the interaction term *Proxy* × *DirOwn* in all specifications of voting outcomes are significantly positive at least at the 10% level. These findings confirm that directors with higher shareholdings at a firm soliciting proxies receive more assent votes, even when filtering out aggregate firm-level satisfaction. The evidence is consistent with the notion that both director ownership and proxy votes are in favor of acquiring assent votes for directors.

**Table 3:** Interaction effects of proxy solicitation and director ownership on voting outcomes, director turnover and spillover effect.

Variable	Non-busy directors					Busy directors				
	(1) %Assent	(2) Excess%Assent	(3) Departure	(4) Chg_Dirship_t	(5) Chg_Dirship_t+1	(6) %Assent	(7) Excess%Assent	(8) Departure	(9) Chg_Dirship_t	(10) Chg_Dirship_t+1
**Proxy × DirOwn**	0.7804*** (3.94)	0.7686*** (4.14)	**0.4471 (0.15)**	**−0.1498 (-0.44)**	**0.2386 (0.42)**	0.2150* (1.67)	0.2439** (2.07)	5.5020*** (2.87)	**0.8342 (0.46)**	**0.2190 (0.08)**
Proxy	−0.0263** (-2.37)	−0.0185* (-1.80)	−0.4711 (-1.59)	0.0195 (0.82)	0.0105 (0.24)	−0.0116 (-0.79)	−0.0049 (-0.41)	−0.3020 (-0.65)	0.1591 (0.94)	0.8891** (2.37)
DirOwn	0.3978*** (5.03)	0.3377*** (4.41)	1.0503 (1.42)	−0.0153 (-0.12)	−0.0947 (-0.37)	0.5131*** (9.54)	0.4000*** (7.48)	−0.9118 (-0.73)	0.3084 (0.52)	0.0591 (0.05)
Size	−0.0284*** (-8.16)	0.0003 (0.12)	−0.0662 (-0.62)	0.0060 (0.53)	0.0115 (0.61)	−0.0352*** (-7.54)	−0.0093** (-2.51)	−0.1667 (-1.14)	0.0414 (0.59)	−0.0914 (-0.66)
StockRet	−0.0133 (-1.10)	−0.0013 (-0.12)	−0.4352 (-1.24)	0.0241 (0.69)	0.0567 (0.69)	−0.0086 (-0.64)	−0.0028 (-0.27)	0.6912** (2.27)	0.2001 (1.29)	−0.0121 (-0.05)
ROA	−0.1798*** (-2.86)	0.0433 (0.93)	−1.9484* (-1.67)	0.1210 (1.02)	0.0899 (0.37)	−0.2711*** (-3.45)	−0.0360 (-0.65)	−2.9256 (-1.52)	−0.1594 (-0.20)	3.4351* (1.78)
InstOwn	−0.0306 (-1.30)	0.0180 (0.92)	−0.3121 (-0.50)	−0.1351** (-2.30)	−0.1705* (-1.75)	−0.0769** (-2.30)	−0.0090 (-0.32)	1.0088 (1.13)	−0.4507 (-1.40)	−0.8015 (-1.47)
Tenure	0.0051*** (6.57)	0.0056*** (6.79)	0.0054 (0.82)	−0.0075*** (-5.21)	−0.0096*** (-3.89)	0.0063*** (10.99)	0.0070*** (13.55)	−0.0614*** (-2.81)	−0.0426*** (-7.84)	−0.0753*** (-5.43)
Male	−0.0236** (-2.26)	−0.0281*** (-2.82)	0.3621 (1.23)	−0.0308 (-1.27)	−0.0162 (-0.29)	−0.0133 (-0.74)	−0.0102 (-0.61)	0.5294 (0.71)	−0.0826 (-0.55)	−0.4146 (-1.13)
MasterAbv	−0.0517*** (-5.61)	−0.0434*** (-4.95)	0.4988** (2.44)	0.0347 (1.34)	0.1138*** (2.84)	−0.0396*** (-3.45)	−0.0419*** (-3.97)	−0.2990 (-0.93)	−0.0716 (-0.69)	−0.1429 (-0.76)
Intercept	1.2904*** (22.42)	−0.1062** (-2.39)	−4.0096*** (-2.82)	0.0314 (0.19)	0.1823 (0.63)	1.4712*** (19.47)	0.0967 (1.61)	−5.5799** (-2.25)	0.1812 (0.17)	2.8375 (1.27)
N R^2^/Pseudo R^2^	5,888 0.1323	5,888 0.1104	5,886 0.0641	5,656 0.0250	2,628 0.0375	3,680 0.1754	3,680 0.1411	3,680 0.1360	3,561 0.0349	1,596 0.0850

*Notes:* This table reports the regression results for the interaction effect of proxy solicitation and director ownership on the measurements of voting outcomes, director turnover and spillover effect, including %Assent, Excess%Assent, Departure, Chg_Dirship_t, and Chg_Dirship_t+1, for non-busy and busy directors. Busy directors concurrently hold three or more directorship appointments at different publicly traded firms. The estimated coefficient of interest is the interaction term of Proxy × DirOwn. Proxy is a dummy variable that equals 1 if proxy solicitation is made in a director election at a firm, and 0 otherwise. DirOwn is the proportion of shares held by an elected director at a firm. Control variables include Size, StockRet, ROA, InstOwn, Tenure, Male, and MasterAbv. All variables are defined in the Appendix. When using Departure as the dependent variable, models are estimated using logistic regressions, otherwise OLS regressions are employed. All specifications include industry fixed effects. *T*-statistics are based on standard errors corrected for heteroskedasticity and clustered at the firm level. ***, **, and * represent 1%, 5%, and 10% significance levels, respectively.

The results regarding career consequences are presented in Models (3) to (5) and (8) to (10) of Table 3. When the dependent variable is *Departure*, the estimate of *Proxy* × *DirOwn* for non-busy directors in Model (3) is insignificant at conventional levels. However, the coefficient of *Proxy* × *DirOwn* for busy directors in Model (8) is positive and statistically significant at the 1% level. Consistent with the arguments by Masulis and Mobbs(2014), we show that in the subsample of busy directors, directors with higher shareholdings at a firm soliciting proxies are more likely to relinquish the directorship at the focal firm because assent arising partially from proxy votes without shareholder voice is less likely to preserve and improve directors’ reputations [6]. When busy directors face a dilemma regarding the allocation of their limited time and energy across their boards, they tend to leave their lower-ranked positions. These findings support Hypothesis 1.

When the dependent variable is *Chg_Dirship_t* or *Chg_Dirship_t+1* in Models (4)–(5) and (9)–(10) (Table 3), the coefficients of *Proxy* × *DirOwn* are statistically insignificant for both subsamples of non-busy and busy directors. Overall, the interaction term between proxy solicitation and director ownership has no effect on changes in the number of other board positions held by the director (regardless of directors being non-busy or busy) over the election year and the subsequent year because proxy votes involve no shareholder voice leading to no spillover effect. Our Hypothesis 2 is thus supported.

Overall, our findings in Table 3 indicate that shareholder voice as expressed through votes in director elections is related to director careers [7,24]. Reputation is an important mechanism for motivating outside directors [1]. Outside directors make an effort to protect their reputation by exiting companies whose reputation is helpless [6,[14], [15], [16]]. We provide evidence to highlight the reputation influence through votes on directors’ job mobility.

### 4.3 Two-stage least squares analysis

To minimize endogeneity, we executed 2SLS analysis. This approach alleviates the endogeneity biases that can be attributed to measurement errors, reverse causality, and unobserved heterogeneity. *ProxyMean*, the instrumental variable, is the mean value of *Proxy* in the same industry and the same year at the director level, excluding the directors at the focal firm concerned. Firms are more likely to follow the decision of proxy solicitation in a director election at the annual general meeting, aligning with their industry peers, and are less likely to be correlated closely with directors’ career consequences.

The results of the 2SLS analysis are shown in Table 4. Model (1) is the first-stage regression, where *Proxy* is the dependent variable. As expected, the coefficient of *ProxyMean* is positive and highly significant. Models (2)–(7) are the second-stage regressions, in which *Departure*, *Chg_Dirship_t*, and *Chg_Dirship_t+1* are the dependent variables. We created an interaction term by multiplying *Proxy* instrumented from the first stage by *DirOwn*. All the results are consistent, showing that the coefficient of the interaction term is statistically insignificant for non-busy directors in Model (2) but positively significant at the 1% level for busy directors in Model (5). Busy directors are inclined to depart from their lower-ranked positions. Models (3)–(4) and (6)–(7) report that the coefficients of the interaction term are insignificant for both non-busy and busy directors. Higher assent votes arising partially from proxy votes without shareholder voice result in no spillover effect. Altogether, we find that the main results hold. The 2SLS approach is substantially less vulnerable to endogeneity. Therefore, our results are not driven by endogeneity.

**Table 4:** Interaction effects of proxy solicitation and director ownership on director turnover and spillover effect: 2SLS analysis.

Variable	First stage	Second stage					
		Non-busy directors			Busy directors		
	(1) Proxy	(2) Departure	(3) Chg_Dirship_t	(4) Chg_Dirship_t+1	(5) Departure	(6) Chg_Dirship_t	(7) Chg_Dirship_t+1
ProxyMean	5.4022*** (9.86)						
**Proxy (Instrumented)** × DirOwn		**−1.3793 (-0.40)**	**−0.2775 (-0.49)**	**−0.3853 (-0.38)**	11.1448*** (2.71)	**−5.5166 (-0.98)**	**5.3594 (1.12)**
Proxy (Instrumented)		−1.7194** (-2.23)	0.2137*** (3.23)	0.0672 (0.45)	0.1147 (0.11)	3.0767*** (3.59)	1.1942 (1.14)
DirOwn		1.7058 (1.28)	0.0352 (0.19)	0.0313 (0.10)	−2.7614 (-1.40)	2.7920* (1.82)	−1.3620 (-0.92)
Size	0.3891*** (5.76)	0.0157 (0.14)	−0.0079 (-0.70)	0.0077 (0.37)	−0.2107 (-1.25)	−0.0680 (-0.53)	−0.1302 (-0.79)
StockRet	0.0579 (0.30)	−0.3819 (-1.13)	0.0237 (0.68)	0.0558 (0.67)	0.7232** (2.33)	−0.9617** (-2.41)	−0.0129 (-0.05)
ROA	−4.1736*** (-3.77)	−2.9474** (-2.29)	0.2457* (1.89)	0.1169 (0.44)	−2.6156 (-1.31)	4.5918** (2.12)	3.7996* (1.71)
InstOwn	−4.6493*** (-9.83)	−1.4045 (-1.64)	0.0242 (0.33)	−0.1309 (-0.86)	1.6518 (1.52)	2.2254** (2.20)	−0.4514 (-0.46)
Tenure	−0.0013 (-0.40)	0.0052 (0.81)	−0.0074*** (-5.16)	−0.0095*** (-3.88)	−0.0613*** (-2.81)	0.0471*** (3.83)	−0.0754*** (-5.43)
Male	0.1002 (1.06)	0.3762 (1.29)	−0.0341 (-1.41)	−0.0161 (-0.29)	0.5437 (0.72)	−0.2709 (-0.71)	−0.4572 (-1.24)
MasterAbv	0.2091*** (2.82)	0.5530*** (2.73)	0.0289 (1.14)	0.1116*** (2.77)	−0.3283 (-1.04)	−0.5045** (-2.03)	−0.1586 (-0.86)
Intercept	−6.6451*** (-6.41)	−4.3061*** (-3.08)	0.1039 (0.63)	0.2029 (0.71)	−5.3867** (-2.13)	−3.1406 (-1.54)	3.1604 (1.36)
N	9,568	5,886	5,656	2,628	3,680	3,680	1,596
Pseudo R^2^ / R^2^	0.3790	0.0666	0.0264	0.0375	0.1386	0.0350	0.0810

*Notes:* This table reports the regression results for the interaction effect of proxy solicitation and director ownership on the measurements of director turnover and spillover effect, including Departure, Chg_Dirship_t, and Chg_Dirship_t+1, for non-busy and busy directors, using two-stage least squares (2SLS) regressions. Busy directors concurrently hold three or more directorship appointments at different publicly traded firms. In the first-stage, we estimated the probability of Proxy based on the instrumental variable, ProxyMean, and the set of control variables in Table 3. We then used the expected probability of Proxy, obtained from the first-stage regression, as explanatory variables in the second-stage regression. ProxyMean is the mean value of Proxy in the same industry and the same year at the director level, excluding the directors at the focal firm concerned. All variables are defined in the Appendix. When using Proxy and Departure as the dependent variables, models are estimated using logistic regressions; otherwise, OLS regressions are employed. All specifications include industry fixed effects. *t*-statistics are based on standard errors corrected for heteroskedasticity and clustered at the firm level. ***, **, and * represent 1%, 5%, and 10% significance levels, respectively.

### 4.4 Propensity score matching analysis

Our previous discussion shows consistent findings. However, the results may have model misspecification or omitted variable bias, which may violate the assumptions of the OLS model. To mitigate such concerns, we employed PSM analysis and created a closely matched sample to check whether directors at firms with and without proxy solicitation differed from one another in terms of career consequences. We classified directors at firms with proxy solicitation as our treatment group. For each director in the treatment group, we used PSM, in which we utilized the nearest neighbor with replacement matching, to identify a control group based on all the control variables such as *Size*, *StockRet*, *ROA*, *InstOwn*, *Tenure*, *Male*, and *MasterAbv* in the regression analysis. Thus, our treatment and control groups are nearly identical across all dimensions.

Table 5(A) reports the univariate mean comparisons between treatment and control groups’ characteristics and their corresponding *t*-statistics. The results demonstrate that the average values of the matching variables are qualitatively similar across the treatment and control groups. We then performed PSM regression using the post-matched sample in Table 5(B). The results indicate that the interaction term between proxy solicitation and director ownership has a positive effect on director turnover for busy directors, but not for non-busy directors. The interaction term between proxy solicitation and director ownership has no effect on the net change in the number of external directorships for both non-busy and busy directors. These findings are consistent with our baseline results, which are unlikely confounded by endogeneity.

**Table:** 

Panel B: PSM regression						
Variable	Non-busy directors			Busy directors		
	(1) Departure	(2) Chg_Dirship_t	(3) Chg_Dirship_t+1	(4) Departure	(5) Chg_Dirship_t	(6) Chg_Dirship_t+1
Proxy × DirOwn	1.1843 (0.34)	−0.0234 (-0.05)	0.1567 (0.22)	5.5400** (2.19)	0.9246 (0.43)	−0.8181 (-0.23)
Proxy	−0.5065 (-1.58)	0.0072 (0.28)	0.0077 (0.15)	−0.2199 (-0.46)	0.2810 (1.20)	1.2390** (2.26)
DirOwn	−0.0964 (-0.07)	−0.0812 (-0.31)	0.0808 (0.15)	−0.9988 (-0.51)	0.3736 (0.36)	1.1972 (0.49)
Size	−0.1036 (-0.68)	0.0174 (1.05)	0.0252 (1.08)	−0.0942 (-0.45)	0.0344 (0.65)	−0.1838 (-1.46)
StockRet	−0.6527 (-1.37)	−0.0175 (-0.39)	0.0578	1.1715*** (2.60)	0.0326 (0.17)	−0.1032 (-0.31)
ROA	−1.5107 (-0.78)	0.1429 (0.85)	0.2521 (0.80)	−4.1878 (-1.24)	0.4359 (0.42)	3.8744 (1.35)
InstOwn	0.2912 (0.32)	−0.1526* (-1.93)	−0.2623* (-1.88)	1.6237 (1.18)	−0.5227 (-1.48)	−1.2734* (-1.70)
Tenure	0.0084 (1.46)	−0.0081*** (-4.15)	−0.0107*** (-3.06)	−0.0515** (-1.97)	−0.0497*** (-7.68)	−0.0880*** (-4.51)
Male	0.0533 (0.16)	−0.0103 (-0.36)	−0.0478 (-0.65)	0.6968 (0.67)	−0.0475 (-0.32)	−0.6609* (-1.67)
MasterAbv	0.6200** (2.24)	0.0228 (0.81)	0.0867* (1.72)	−0.2808 (-0.79)	0.0127 (0.10)	−0.0733 (-0.31)
Intercept	−5.5373*** (-2.67)	−0.1350 (-0.57)	0.0238 (0.07)	−7.9281** (-2.43)	0.6564 (0.80)	4.3615** (2.08)
N	3858	3,697	1,689	2,291	2,217	998
Pseudo R2/R2	0.0858	0.0328	0.0650	0.1812	0.0486	0.1100

*Notes:* This table reports the regression results for the interaction effect of proxy solicitation and director ownership on the measurements of director turnover and spillover effect, including Departure, Chg_Dirship_t, and Chg_Dirship_t+1, for non-busy and busy directors using propensity score matching (PSM). Busy directors concurrently hold three or more directorship appointments at different publicly traded firms. We define directors at firms with proxy solicitation as belonging to the treatment group. Control directors are matched directors at firms without proxy solicitation. Control directors are matched using PSM (nearest director with replacement) on the same control variables as presented in Table 3. Panel A shows the univariate mean comparison between treatment and control directors' characteristics and their corresponding *t*-statistics. Panel B shows the results of PSM regressions on the matched sample. All variables are defined in the Appendix. When using Departure as the dependent variable, models are estimated using logistic regressions; otherwise, OLS regressions are employed. All specifications include industry fixed effects. *t*-statistics are based on standard errors corrected for heteroskedasticity and clustered at the firm level. ***, **, and * represent 1%, 5%, and 10% significance levels, respectively.

**Table 5:** Interaction effects of proxy solicitation and director ownership on director turnover and spillover effect: PSM analysis.

Panel A: Comparison of treatment and control directors						
Variable	N	Treated	N	Control	Differences	*t*-statistics
Size	3075	16.2194	3,075	16.2437	−0.0243	−0.59
StockRet	3075	0.0910	3,075	0.0919	−0.0009	−0.10
ROA	3075	0.0770	3,075	0.0769	0.0001	0.06
InstOwn	3075	0.4168	3,075	0.4230	−0.0062	−1.20
Tenure	3,075	11.6467	3,075	11.7671	−0.1204	−0.41
Male	3,075	0.8715	3,075	0.8657	0.0059	0.68
MasterAbv	3,075	0.5707	3,075	0.5545	0.0163	1.29

## 5 Conclusion

This study contributes to the recent literature by exploring the joint effect of proxy solicitation and director ownership on directors' career consequences [7,24,33]. Prior studies show that shareholder voice of dissatisfaction with directors has adverse effects on directors' careers. This study provides complementary evidence to support this theory. Assent votes partially resulting from proxies without shareholder voice increase the likelihood of departures for directors with higher ownership at firms soliciting proxies in the subsample of busy directors, but not in the subsample of non-busy directors. Consistent with Masulis and Mobbs (2014), the evidence illustrates that each directorship position provides unequal incentives for busy directors [6]. Considering their limited time and energy, busy directors prefer to stay in prestigious board seats compared with those deemed less valuable. Since higher assent votes partially lacking shareholder voice do not build additional reputation, no spillover effect is generated for directors with greater ownership at firms soliciting proxies in both subsamples of non-busy and busy directors. We conclude that proxy votes in director elections carrying no shareholder voice do have implications on directors' careers. Busy directors distribute effort and resources to their directorships based on each board seats’ relative contribution to their reputation.

The limitation of this research is that based on the available databases in Taiwan, we could not identify a director election as uncontested or contested, like done in the study by Aggarwal et al. (2019) [7]. Therefore, we examined both uncontested and contested director elections. However, we believe that this limitation will not affect our contribution to the literature as shareholder voice implied in votes should matter in both uncontested and contested elections. Furthermore, contested director elections are trivial because the proportion is very small every year.

## Production notes

### Author contribution statement

Tai-Wei Zhang; Yun-Chi Lee: Conceived and designed the experiments; Performed the experiments; Analyzed and interpreted the data; Contributed reagents, materials, analysis tools or data; Wrote the paper.

### Funding statement

Professor Yun-Chi Lee was supported by National Science and Technology Council in Taiwan [NSTC 111-2410-H-031-095- and NSTC 110-2410-H-031-084-].

### Data availability statement

Data will be made available on request.

### Declaration of interest’s statement

The authors declare no conflict of interest.

## Figures and Tables

**Table:** 

Variable	Description
Panel A: Voting outcomes and career consequences	
%Assent	The number of votes received by an elected director divided by the total votes, which is the total shares represented by shareholders present in person or by proxy at a shareholder meeting at a firm.
Firm%Assent	The average percentage of assent votes at the firm level, which is the mean of *%Assent* across all directors at a firm in an election event.
Excess%Assent	The *%Assent* for a director minus the firm-level average, *Firm%Assent*.
Departure	A dummy variable that equals 1 if a director of a firm is no longer on the board within 1 year following a particular shareholder meeting date in which they sought reelection at the firm, and 0 otherwise.
Dirship_t-1	The total number of directorships held by a director at the end of the year prior to a director election, excluding the focal firm at which the director is up for election.
Dirship_t	The total number of directorships held by a director at the end of the year in which a director election takes places, excluding the focal firm at which the director is up for election.
Dirship_t+1	The total number of directorships held by a director at the end of the year following a director election, excluding the focal firm at which the director is up for election.
Chg_Dirship_t	The one-year net change in the number of outside directorships held by a director, which is calculated as *Dirship_t* minus *Dirship_t-1*.
Chg_Dirship_t+1	The two-year net change in the number of outside directorships held by a director, which is calculated as *Dirship_t+1* minus *Dirship_t-1*.
Panel B: Main variables	
Proxy	A dummy variable that equals 1 if proxy solicitation was made in a director election at a firm, and 0 otherwise.
DirOwn	The proportion of outstanding shares held by a director of the board at month-end prior to a director election.
Busy director	Directors who hold at least three directorship seats at different publicly traded firms.
Non-busy director	Directors who hold at least one but fewer than three directorship seats at different publicly traded firms.
Panel C: Control variables	
*Firm characteristics*	
Size	The natural logarithm of the book value of total assets at the end of the year prior to a director election.
StockRet	The 12-month buy-and-hold return over the year prior to a director election.
ROA	The ratio of earnings before interest, taxes, depreciation, and amortization (EBITDA) to average total assets at the end of the year prior to a director election.
InstOwn	The proportion of outstanding shares held by institutional owners at year-end prior to a director election.
*Director characteristics*	
Tenure	The length of time a director has served on the current board in years.
Male	A dummy variable that equals 1 if a director is male, and 0 otherwise.
MasterAbv	A dummy variable that equals 1 if a director is reported in the TEJ as having a master's degree or above, and 0 otherwise.
